# MRI-defined patterns of infiltration and outcome in patients with glioblastoma

**DOI:** 10.1093/noajnl/vdaf114

**Published:** 2025-07-11

**Authors:** Katrin Scheu, Edith Vandieken, Katharina Hense, Katharina Rosengarth, Tareq Haedenkamp, Markus Lenglinger, Elisabeth Bumes, Ralf Linker, Martin Proescholdt, Nils Ole Schmidt, Markus J Riemenschneider, Christina Wendl, Isabel Wiesinger, Peter Hau

**Affiliations:** Department of Neurology, University Hospital Regensburg, Regensburg, Germany; Department of Neurology, University Hospital Regensburg, Regensburg, Germany; Department of Neurosurgery, University Hospital Regensburg, Regensburg, Germany; Department of Neurosurgery, University Hospital Regensburg, Regensburg, Germany; Wilhelm Sander-NeuroOncology Unit, University Hospital Regensburg, Regensburg, Germany; Department of Neurology, University Hospital Regensburg, Regensburg, Germany; Wilhelm Sander-NeuroOncology Unit, University Hospital Regensburg, Regensburg, Germany; Department of Neurology, University Hospital Regensburg, Regensburg, Germany; Wilhelm Sander-NeuroOncology Unit, University Hospital Regensburg, Regensburg, Germany; Department of Neurology, University Hospital Regensburg, Regensburg, Germany; Department of Neurology, University Hospital Regensburg, Regensburg, Germany; Department of Neurosurgery, University Hospital Regensburg, Regensburg, Germany; Department of Neurosurgery, University Hospital Regensburg, Regensburg, Germany; Department of Neuropathology, University Hospital Regensburg, Regensburg, Germany; Center for Neuroradiology, Institute for Diagnostic Radiology, University Hospital Regensburg, Regensburg and District County Hospital Regensburg, Regensburg, Germany; Center for Neuroradiology, Institute for Diagnostic Radiology, University Hospital Regensburg, Regensburg and District County Hospital Regensburg, Regensburg, Germany; Wilhelm Sander-NeuroOncology Unit, University Hospital Regensburg, Regensburg, Germany; Department of Neurology, University Hospital Regensburg, Regensburg, Germany

**Keywords:** glioblastoma, infiltration, prognostic markers, magnetic resonance imaging, survival

## Abstract

**Background:**

Glioblastoma is a rare primary tumor of the brain. Infiltration of glioblastoma into the brain parenchyma may influence prognosis. We therefore aimed to investigate possible influences of distinct patterns of MRI-defined infiltration on the prognosis.

**Methods:**

We performed a retrospective analysis of sequential patients with glioblastoma between April 2005 and December 2017. Patient data were collected from the hospital data management system, MRI images from the hospital PACS and from cooperative radiology units. Patients were divided into subgroups based on the tumor growth pattern (frame-like, palisade-like, infilling). The impact of various factors on overall survival and progression-free survival was then calculated and compared between the groups.

**Results:**

259 patients were included. Of the 258 evaluable patients, 117 showed a palisade-like infiltration, 98 were classified as non-infiltrating frame-like, and 43 as infilling dense-solid. Standard prognostic factors aligned to published data. In multivariate analysis, no significant influence of palisade-like growth on overall survival and progression-free survival could be detected. In Cox regression analyses, we found a significant effect for overall survival in palisade-like tumors in the univariate analysis (OR 1.354, 95% CI 1.032–1.776, *P* = .029).

**Conclusion:**

We show here a possible correlation of MRI-based infiltration patterns and survival in patients with glioblastoma. Our results correspond well to published literature that shows that certain subtypes of glioblastoma exhibit an enhanced invasion pattern and decreased survival. Our results should be verified in a prospective setting in a large patient cohort and by using automated methods for the classification of infiltration patterns in glioblastoma.

**Key Points**

- MR morphological growth patterns, namely a palisade-like growth pattern, may negatively relate to overall survival.
- Further studies are needed to validate our results in larger cohorts, also using automated methods for the investigation of infiltration.

**Importance of the Study**

The prognosis of glioblastomas remains poor and differs significantly between individual patients. Tumor cell infiltration is a dominant biological principal in glioblastoma and correlates to the mesenchymal glioblastoma subtype. Standard magnetic resonance imaging (MRI)-based morphological imaging data are collected in regular intervals during the treatment course and are able to monitor tumor infiltration. This paper strongly suggests a possible correlation of MRI-based infiltration patterns and survival in patients with glioblastoma by use of a non-invasive, simple, and cost-effective MRI-based methodology. We thereby propose an additional prognostic factor, namely invasion pattern, in glioblastoma patients that may be able to refine the prognosis of glioblastoma.

Glioblastomas, IDH wild-type (CNS WHO Grade 4) are malignant primary brain tumors originating from glial cells^1^ They account for 50.1% of primary malignant brain tumors, with an incidence of about 3 per 100,000 people/year.^2^ Their high aggressiveness and complexity lead to a particularly poor prognosis. Even with advanced therapies, the median overall survival remains low at about 21 months and less than 7% of the patients are alive after 5 years of diagnosis.^2^

Survival of patients with glioblastoma depends on well-established prognostic factors, such as age, sex, the functional status after primary resection (measured by the Karnofsky performance status scale (KPS) or ECOG)^3–5^ as well as methylation of the MGMT (O6-methylguanine-DNA-methyltransferase)-promoter.^6^ In the WHO classification of 2016, the IDH mutational status was also defined as a defining and prognostic factor for IDH-mutant glioblastoma.^1^ Recently, temporal muscle thickness has been added as another potential prognostic factor;^7^ however, it is not fully acknowledged as a standard prognostic factor yet.

The pathophysiology of glioblastoma has not been completely understood so far. Glioblastomas show almost no distant metastasis.^8^ Instead, tumor cells can spread using pre-existing structures such as blood vessels, white matter tracts, and the subarachnoid space.^9^ Due to remodeling in the cytoskeleton and the extracellular matrix, tumor cells can thereby cross tissue barriers and migrate into surrounding tissue.^10^ On a molecular level, an infiltrative phenotype has been convincingly connected to the mesenchymal subtype of glioblastoma, which is also connected to inferior survival.^11^ However, a clear relation of a macroscopic infiltrative subtype with decreased overall survival has not been established yet.

The macroscopic infiltrative pattern of glioblastoma can be observed by imaging modalities as magnetic resonance imaging (MRI)^12,13^ and positron emission tomography.^14^ Recently, efforts have been made to determine the prognosis of patients more precisely using diffusion MR imaging metrics.^15^ In the context of radiogenomics, attempts were also made to link various image morphological properties with molecular properties.^16^ However, these methods are not yet used in routine clinical practice.

There are several published studies that have shown clear MRI morphological patterns. For example, tumor size,^17^ surface regularity, and a contrast-enhancing rim were defined as image morphological characteristics.^18^ So far, there have been no convincing studies on whether certain patterns of infiltration in MRI are directly related to overall survival. In this study, we therefore divided patients into three predefined cohorts with a distinct image morphology. We hypothesized that distinct infiltration patterns would relate to overall survival, with a more infiltrative pattern showing inferior survival.

## Materials and Methods

### Study Design and Patients

We collected clinical and radiographic data from patients newly diagnosed with glioblastoma between April 22, 2005 and December 20, 2017 at a single academic neuro-oncologic center in Germany (University Hospital Regensburg). Patients were drawn from the local tumor registry (Onkostar, IT Choice Software AG, Karlsruhe, Germany), and data were derived from the hospital patient management system (ISH-Med, SAP Deutschland SE & Co. KG, Waldorf, Germany) and the respective PACS systems (Syngo, Siemens Healthineers AG, Forchheim, Germany). Radiographic data were collected as DICOM (Digital Imaging and Communications in Medicine) files from the local PACS or cooperating radiology units.

The following inclusion criteria were predefined: Adult patients with newly diagnosed with glioblastoma according to the WHO classification of 2007; availability of clinical, prognostic, and survival data as well as radiological data available. At least one contrast-enhanced MRI scan at the time of diagnosis and before intervention or any therapy was required for inclusion. If a recurrence had occurred, the MRI at recurrence was also recorded. Minimum requirements for MRI were FLAIR and a 3D-T1 sequence after contrast application. The main reasons for study exclusion were incomplete imaging data at initial diagnosis, incomplete clinical data, lack of histological confirmation, as well as incomplete follow-up. Glioblastomas without contrast enhancement were also excluded.

All clinical data were obtained within the framework of routine clinical assessments. In addition to demographic data, such as age, sex, the extent of resection, KPS before and after resection and tumor localization, dates of resection, progression, and overall survival were recorded. Data on MGMT promoter methylation status and IDH mutational status were added by querying the local neuropathology database. After the compilation of the cohort, patients were evaluated alphabetically.

All data were collected in a pseudonymized format. The response assessment was conducted during independent, interdisciplinary tumor board meetings that included a dedicated neuroradiologist in consensus with clinicians who provided the clinical data, therefore taking into account both radiological and clinical aspects.

The study was approved by the local ethics committee of the University Regensburg (application number: 21-2261-104). Due to the retrospective design of the study and as only data from the framework of routine clinical assessments were used, no patient informed consent was raised in accordance with ethics and data protection guidelines in Germany.

### Imaging Review

Archived MRI images were independently reviewed to determine the pattern of infiltration and for validation of tumor recurrence by two experienced neurology residents (K.S., E.V.) together with a neuroradiology specialist (I.W.) in consensus.

**Figure 1.**
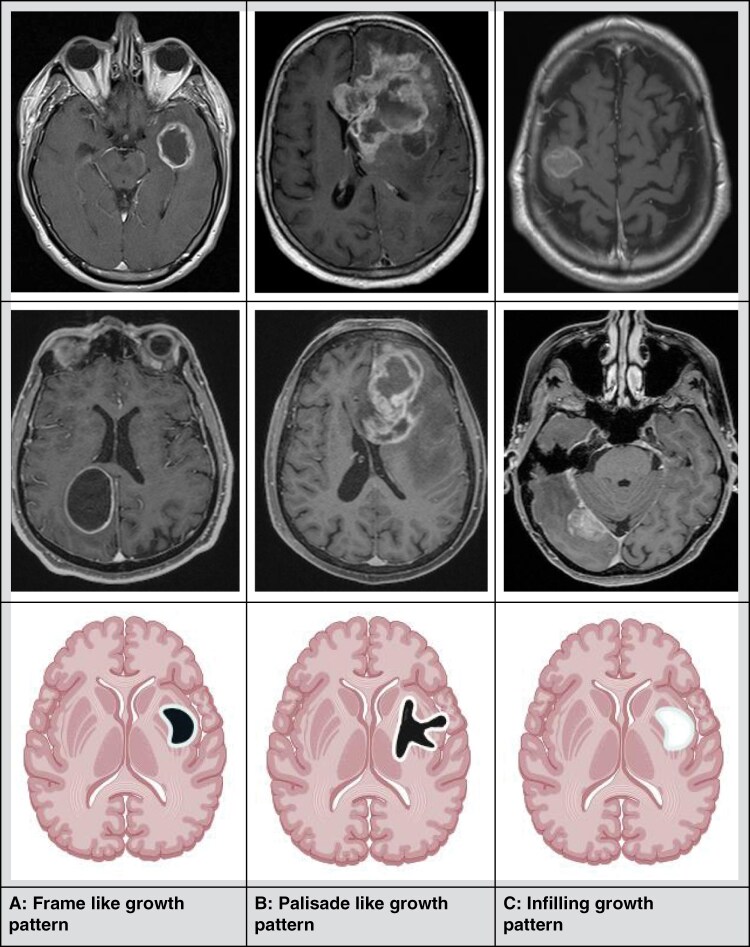
Definition of infiltration patterns. 1A, frame like, 1B, palisade like, and 1C, infilling.

MRI-based tumor-specific data included the total number of contrast-enhancing foci, the number of contrast-enhancing foci > 1 cm, localization of the main tumor (hemisphere and lobe, cross-hemisphere), geometric shape of tumor enhancement (round, oval or polylobulated), the shape of the contrast enhancement pattern of the tumor (frame-like, palisade-like, infilling), degree of macrovascularisation within the necrosis (visible vessels in the necrosis area), maximum diameter of largest contrast agent accumulation in mm, maximum diameter of necrosis, thickness of accumulating fringe (measured at 3 points, mean value), and demarcation of tumor enhancement to adjacent tissue (sharp or diffuse). These data were classified into three typical growth patterns. The frame-like pattern is thereby characterized by a sharp demarcation, a fine contrast enhancement line, and homogeneous thickness of the contrast-enhancing rim. Polylobulated growth patterns with inhomogeneous thickness of the rim, some with sharp and some with blurred borders, were described as palisade-like. In the case of infilling growth patterns, a homogeneous contrast enhancement throughout the tumor mass was described (Figure 1).

Treatment response was evaluated as per the updated response assessment criteria for high-grade gliomas^19,20^ using archived MRI scans performed at 8- to 12-week intervals.

### Statistics

All primary source data were processed into Excel datasheets (Microsoft Office, Microsoft, Redmond, USA), verified by double check, cleared, and transferred to SPSS version 28 (IBM, Armonk, New York, USA) for statistics.

To identify relevant factors influencing overall survival (OS) and progression-free survival (PFS), univariate analyses of variance (ANOVA) were performed for the individual prognostic factors age (< 70 years or ≥ 70 years), sex (male, female, or diverse), extent of resection (biopsy, subtotal resection, or gross total resection), KPS (< 80 or ≥ 80) after resection, IDH mutation (mutated or wildtype), MGMT promoter methylation status (unmethylated or methylated), and infiltration pattern (frame-like, palisade-like, or infilling). In addition, Pearson/Spearman correlations with OS and PFS were calculated for the factors age, KPS score, and MGMT promoter methylation.

To analyze interaction effects between individual prognostic factors, a multivariate ANOVA was also calculated for overall survival. Due to the retrospective nature of the patient data and the resulting exclusion of incomplete patient cases from the analysis, the analysis was limited to the most relevant factors age, extent of resection, and MGMT promoter methylation status as well as infiltration pattern. These were also used as covariates in an additional evaluation of the infiltration patterns to eliminate their influence on overall survival.

In addition to the p values, effect sizes (Cohen’s *d*) were calculated for all analyses of variance.

Survival analyses were calculated using univariate as well as multivariate Cox regression analyses for each factor and plotted using the Kaplan–Meyer method.

## Results

### Patients and Demographic Data

A total of 258 patients were evaluated (Table 1). The mean age of patients at initial diagnosis was 62.96 years. Of all patients treated, 13.8% of patients underwent biopsy, 57.5% underwent partial resection, and 25.7% underwent complete resection. The KPS was 90 (range 10–100) after surgery.

**Table 1.** Patient Characteristics

	All patients (*N* = 258)
**Age, years**	
Mean (SD)	62.96 (11.86)
Median (range)	64 (24–87)
**Sex, No. (%)**	
Female	115 (42.9)
Male Diverse	153 (57.1) 0 (0%)
**Extent of resection, No. (%)**	
Biopsy	37 (13.8)
Partial resection	144 (57.5)
Gross total resection	69 (25.7)
Unknown	8 (3.0)
**Karnofsky Performance Status** Before resection, median (range)	90.0 (10–100)
After resection, median (range)	90.0 (10–100)
**MGMT Promoter Methylation (> 4%)**	
Yes	85 (31.7)
No	121 (45.2)
Unknown	52 (23.1)
**IDH 1/2 -Mutation**	
Yes	8 (3.0)
No	157 (58.6)
Unknown	103 (38.4)
**Infiltration pattern**	
Frame-like	100 (37.6)
Palisade-like	121 (45.5)
Infilling	45 (16.9)

MGMT promoter methylation was detected in 31.7% of patients, well corresponding to the published literature. In our study population, 3.0% of patents had an IDH 1/2 mutation, whereas 58.6% were not mutated. Data on IDH mutation were missing for the residual 38.4% of patients, but histological criteria for the diagnosis glioblastoma were fulfilled according to the WHO 2016 classification.

Using our archived MRI data, we found a frame-like pattern in 100 patients (37.6%), a palisade-like pattern in 121 patients (45.5%), and an infilling pattern in 45 patients (16.9%).

### Prognostic Factors, Progression-free, and Overall Survival

To investigate the influence of individual prognostic factors on overall survival (OS), a univariate analysis of variance (ANOVA) was performed for each of the factors age, sex, extent of resection, Karnofsky Performance Scale (KPS) after resection, MGMT promoter methylation status, IDH mutation, and infiltration pattern (Supplementary Table 1). There were statistically significant differences in overall survival regarding the prognostic factors of age, extent of resection, KPS score after surgery, and MGMT promoter methylation status. Correlation analyses revealed significant correlations between OS and age and KPS after surgery but not for the degree of MGMT promoter methylation.

To investigate whether the named prognostic factors had an impact on progression-free survival (PFS), additional univariate analyses were calculated (Supplementary Table 2). There were significant differences in PFS with regard to the extent of resection and MGMT promoter methylation status; however, no effect was found for the other prognostic factors including KPS. Correlation analyses showed no significant correlations.

To investigate our primary hypothesis, one-way ANOVAs were calculated for the imaging growth pattern in relation to overall survival and PFS (Table 2). We found a trend regarding overall survival (*P* = .080) and no significant difference regarding PFS (*P* = .745).

**Table 2.** Influence of Infiltration Patterns on Overall Survival and Progression-free Survival (univariate analysis, ANOVA)

Analysis	Characteristics	*n*	Mean OS (in weeks)	*P*	Cohen’s *d*
**Overall survival**	Frame-like	98	89.510	.080	.2857
	Palisade-like	117	69.516		
	Infilling	43	75.040		
**Progression-free** **survival**	Frame-like	77	47.832	.745	.1097
	Palisade-like	75	45.427		
	Infilling	29	40.703		

### Multivariate Analysis

To correct for statistically significant influences, a multivariate analysis of variance (ANOVA) was performed using the factors age, extent of resection, MGMT promoter methylation status, and infiltration pattern. The multivariate analysis showed main effects for the factors age (*P* = .015), extent of resection (*P* = .000), and MGMT promoter methylation status (*P* = .011), but not for the factor infiltration pattern (*P* = .398). Furthermore, we investigated possible interactions between the included factors. There were no interaction effects between the individual factors (Supplementary Table 3).

The evaluation of infiltration patterns using a one-way ANOVA with the factors age, extent of resection, and MGMT promoter methylation status as covariates showed significant effects for the three covariates (each *P* < .001), but no significant effect for the factor infiltration pattern (*P* = 0.148) (Supplementary Table 4).

### Overall Survival and Progression-free Survival

In addition to univariate and multivariate analyses of variance for prognostic factors, overall survival and PFS were analyzed using univariate Cox regression analyses for each prognostic factor as well as a multivariate Cox analysis for all factors combined.

Regarding overall survival, we found significant differences in survival for the factors age (*P* < .001), extent of resection (*P* < .001), KPS score after surgery (*P* = .06), and MGMT promoter methylation status (*P* < .001) in the univariate as well as the multivariate analyses (Table 3, Supplementary Figure 2). In the multivariate analyses, longer survival was associated with an age < 70 years (OR 1.878, 95% CI 1.209–2.916, *P* = .005), subtotal (OR.527, 95% CI 0.325–0.855, *P* = .009) or gross total resection (OR 0.343, 95% CI 0.202–0.583, *P* < .001), a KPS score of ≥ 80 (OR 0.537, 95% CI 0.338–0.854, *P* = .009) and a methylated MGMT promoter (OR 0.462, 95% CI 0.310–0.688, *P* < .001).

**Table 3.** Influence of Prognostic Parameters and Imaging Patterns on Overall Survival (Cox regression analysis)

		Univariate Cox regression analysis					Multivariate Cox regression analysis				
Variable	Characteristics	*n*	*P*	OR	Lower 95%-CI	Upper 95%-CI	*n*	*P*	OR	Lower 95%-CI	Upper 95%-CI
**Age**	Age < 70 years	174		1.000			54		1.000		
	Age ≥ 70 years	84	< 0.001	2.018	1.546	2.635	27	.005	1.878	1.209	2.916
**Sex**	Male	148		1.000			50		1.000		
	Female	110	.637	.942	.735	1.207	31	.153	.756	.515	1.110
**Extent of resection**	Biopsy	36		1.000			16		1.000		
	Subtotal resection	150	< 0.001	.523	.362	.755	41	.009	.527	.325	.855
	Gross total resection	64	< 0.001	.391	.259	.591	24	< 0.001	.343	.202	.583
**KPS score after surgery**	< 80	43		1.000			19		1.000		
	≥ 80	134	.006	.615	.435	.871	62	.009	.537	.338	.854
**IDH**	wildtype	150		1.000			76		1.000		
	mutant	7	.382	.712	.332	1.527	5	.428	.687	.272	1.736
**MGMT**	Unmethylated	118		1.000			48		1.000		
	Methylated	80	< 0.001	.584	.436	.782	33	< 0.001	.462	.310	.688
**Infiltration pattern**	Frame-like	98		1.000			31		1.000		
	Palisade-like	117	.029	1.354	1.032	1.776	34	.766	1.071	.681	1.684
	Infilling	43	.277	1.223	.850	1.760	16	.387	1.279	.733	2.231

For progression-free survival, we found a significant effect of the factor “extent of resection” and a trend for MGMT promoter methylation status in both, univariate and multivariate analyses (Table 4). In the multivariate analyses, longer survival was associated with subtotal (OR 0.106, 95% CI 0.041–0.278, *P* < .001) or gross total resection (OR 0.105, 95% CI 0.037–0.299, *P* < .001) and a methylated MGMT promoter (OR 0.421, 95% CI 0.196–0.905, *P* = .027).

**Table 4.** Influence of Prognostic Parameters and Imaging Patterns on Progression-free Survival (Cox regression analysis)

		Univariate Cox regression analysis					Multivariate Cox regression analysis				
Variable	Characteristics	*n*	*P*	OR	Lower 95%-CI	Upper 95%-CI	*n*	*P*	OR	Lower 95%-CI	Upper 95%-CI
**Age**	Age < 70 years	144		1.000			47		1.000		
	Age ≥ 70 years	37	.904	.962	.516	1.795	16	.934	1.038	.426	2.532
**Sex**	Male	106		1.000			39		1.000		
	Female	75	.460	.836	.521	1.344	24	.326	.703	.348	1.420
**Extent of resection**	Biopsy	20		1.000			9		1.000		
	Subtotal resection	105	< 0.001	.176	.086	.360	33	< 0.001	.106	.041	.278
	Gross total resection	52	< 0.001	.186	.086	.402	21	< 0.001	.105	.037	.299
**KPS score after surgery**	< 80	23		1.000			12		1.000		
	≥ 80	104	.949	.976	.470	2.030	51	.354	.650	.261	1.618
**IDH**	wildtype	113		1.000			37		1.000		
	mutant	6	.757	1.176	.421	3.285	26	.357	.537	.143	2.019
**MGMT**	unmethylated	87		1.000			59		1.000		
	methylated	65	.054	6.02	.359	1.009	4	.027	.421	.196	.905
**Infiltration pattern**	Frame-like	77		1.000			27		1.000		
	Palisade-like	75	.767	1.079	.652	1.786	28	.454	1.373	.599	3.143
	Infilling	29	.746	1.128	.544	2.338	8	.187	2.083	.701	6.190

**Figure 2.**
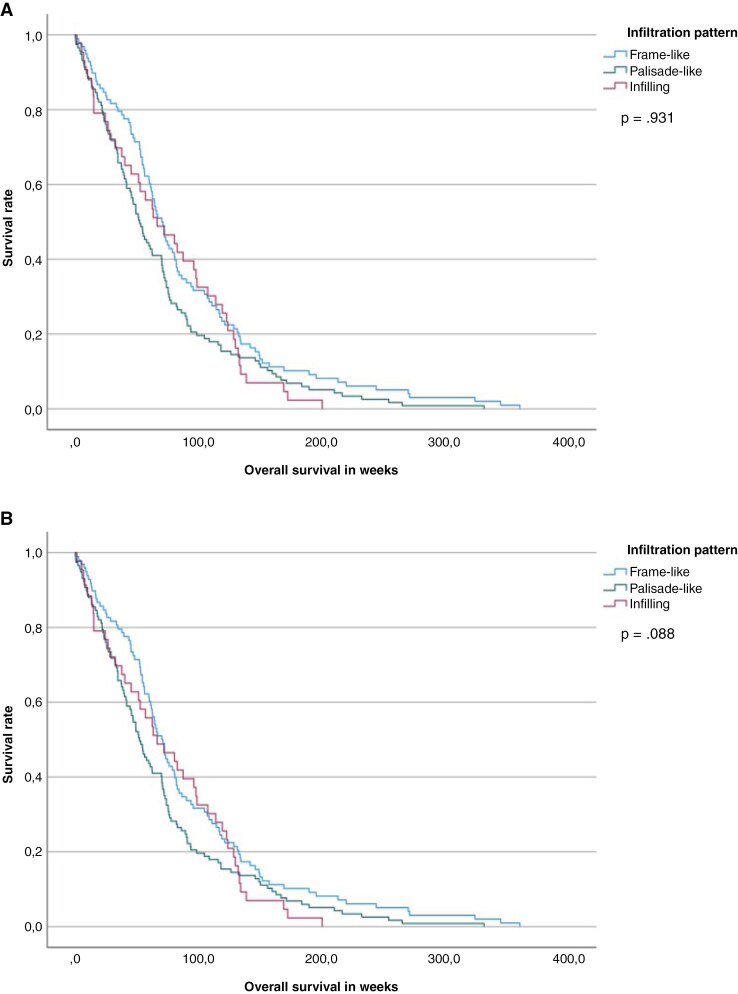
Kaplan Meier survival analysis (overall survival).

Regarding infiltration pattern, we found a significant effect for overall survival in palisade-like tumors in the univariate analysis (OR 1.354, 95% CI 1.032–1.776, *P* = 0.029) and a trend in the Kaplan–Meier survival analysis (89.5 vs. 69.5 weeks; *P* = .08), but no significant effect in the multivariate Cox regression analysis and the Kaplan–Meyer PFS analysis (Table 3, Figure 2).

We also investigated, if a combination of frame-like plus infilling growth patterns vs. palisade-like growth would change the results. Whereas the difference in overall survival gains significance with this approach (*P* = .058 vs. *P* = .080), results become less meaningful in multivariate Cox regression analyses (*P* = .398 vs. *P* = .620).

## Discussion

This study investigated the influence of the morphological tumor invasion pattern on survival of patients with glioblastoma. In summary, our results support the hypothesis that different growth patterns have an impact on the prognosis of patients with glioblastoma. However, whereas effects for overall survival in palisade-like tumors were significant in our univariate analysis and showed and a trend in the Kaplan Meier survival analysis, no significant effect could be shown in the multivariate Cox regression analysis and the Kaplan–Meyer PFS analysis, leaving some room for interpretation.

Our cohort of 258 patients with glioblastoma is representative for such type of tumor. Our univariate analysis of variance showed a significant difference in overall survival for the prognostic factors age, extent of resection, KPS score after surgery, and MGMT promoter methylation status. In the published literature, increased age is associated with shortened overall survival.^4^ Patients with a higher extent of resection,^21^ a high KPS score after surgery^22^, and the presence of MGMT methylation^23^ commonly showed better overall survival. In multivariate analyses, we could show similar effects for the factors age, extent of resection, and MGMT promoter methylation status.

So far, there have only been a few studies with a similar approach. It has been shown that baseline tumor volume may have an effect on overall survival in recurrent GBM.^20^ Pérez-Beteta et al. created a prognosis score including CE rim width, CE volume, surface regularity, and age. Their results suggest that quantitative baseline morphological features in addition to age may be key biomarkers for OS in GBM.^18^ Since they used a different approach in image analyzing and geometrical measuring, these data are not fully comparable to our dataset. Other studies used imaging practices that do not meet current clinical standards, including functional magnetic resonance imaging (fMRI) or magnetic resonance spectroscopy (MRS).^24^ Great progress has also been made in various studies with the attempt to predict molecular characteristics on the basis of MRI data (radiogenomics).^25^ However, to obtain reliable radiophenotypic patterns of specific molecular characteristics and to relate them to clinical outcomes, there are typically not yet sufficiently large data sets available. Together, the use of advanced techniques makes transferability to everyday clinical practice difficult. In contrast, our approach is easily transferable to daily practice, as only MRI sequences from clinical routine were used in this study (T2-FLAIR, T1CE).

Our study has some limitations. First, the study is single-center and retrospective in nature. Second, due to the timing of the data collection (April 2005 to December 2017), the WHO classification of 2007 was used. This means that IDH-positive tumors, which would now be classified as WHO grade 4 astrocytomas, are also part of the cohort. IDH-mutation status was only evaluated in approximately 60% of our population, however, all tumors fulfilled the classical histological criteria for glioblastoma so that, except for the IDH-mutated tumors, the majority would also be classified as glioblastomas according to the 2021 WHO criteria. In addition, a cohort of 258 patients represents a rather small patient population for such type of study. In a larger cohort, the findings that relate to a palisade-like growth pattern might have been even more convincing. Another limitation of this study is that no formal interobserver variability evaluation was done. However, the first assessment of the images was independently done by two readers, and if no consensus was reached, a discussion of the results in between raters was initiated.

The study has also several strengths. First, the used method is simply applicable in daily clinical routine. The necessary MRI data is available for every patient who is treated according to clinical standards, and only morphological images need to be collected to assess the pattern of growth. Furthermore, our method represents a non-invasive and time-sparing approach to predict the prognosis of individual patients.

In order, however, to be able to routinely incorporate the findings of this study into the treatment of patients with glioblastoma, studies with larger cohorts must be carried out to verify our results. To make this possible, the evaluation of image files should be made more efficient, for example through automated measurements. In the future, machine-learning approaches will play an increasingly important role in radiological diagnostics. To train these programs, image morphological criteria are required on the basis of which, for example, a prognosis estimate can be made.^25^ Our study is well suited to fuel into such approaches, with the vision to improve imaging assessments of patients with glioblastoma and to develop more effective treatment strategies afterward.

## Supplementary Material

vdaf114_suppl_Supplementary_Figure_1

vdaf114_suppl_Supplementary_Tables_2-4

vdaf114_suppl_Supplementary_Tables_1_Figures_2

## Data Availability

Data will be made available upon reasonable request.
