# Supplementary figures and images for: MRI-defined patterns of infiltration and outcome in patients with glioblastoma
Source: Neurooncol Adv. 2025 Jul 11;7(1):vdaf114. doi: 10.1093/noajnl/vdaf114 (PMC12365898; doi:10.1093/noajnl/vdaf114)

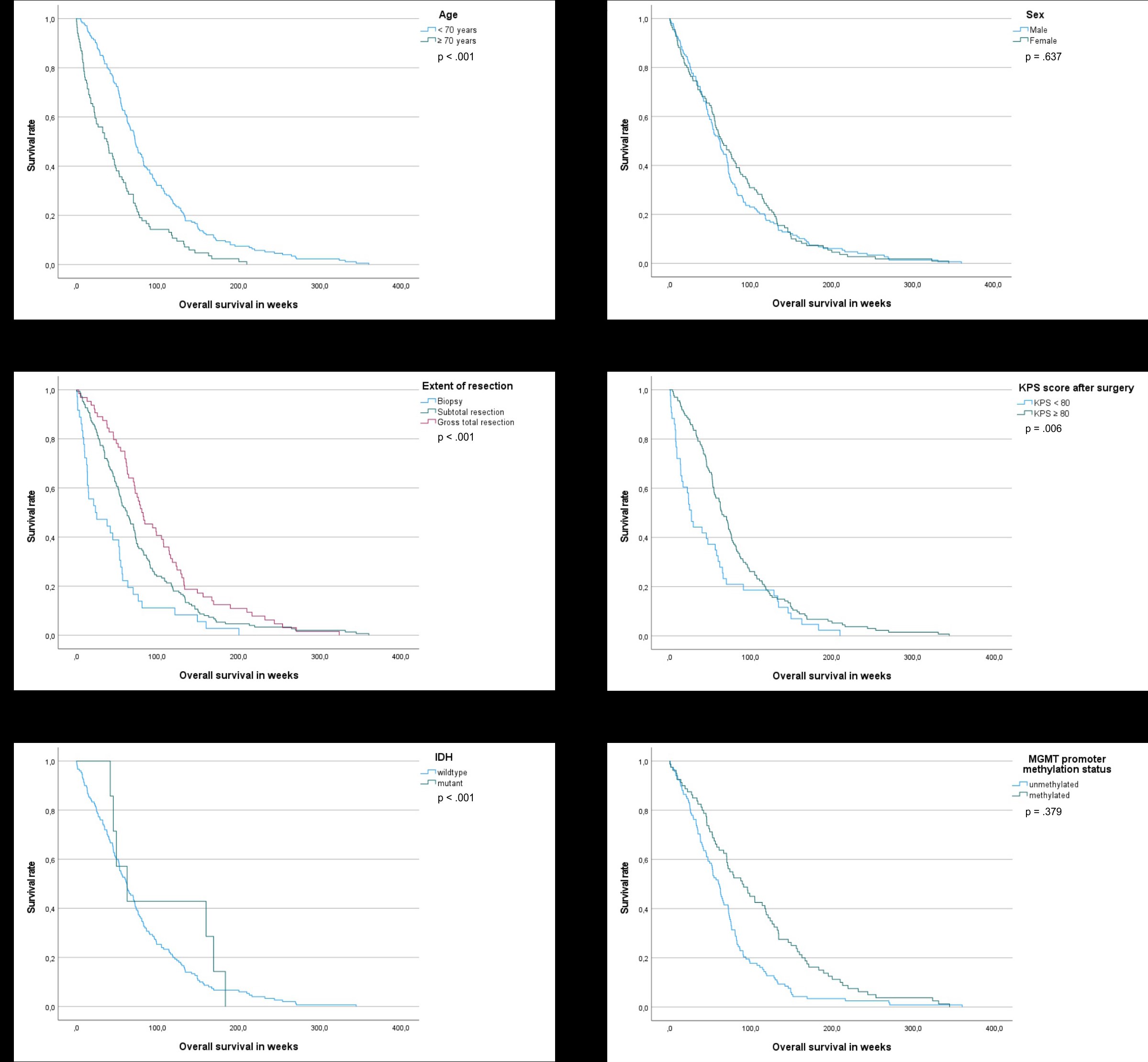

Supplement: vdaf114_suppl_Supplementary_Figure_1 [file vdaf114_suppl_supplementary_figure_1.jpeg]
