# Supplementary material for: MRI-defined patterns of infiltration and outcome in patients with glioblastoma
Source: Neurooncol Adv. 2025 Jul 11;7(1):vdaf114. doi: 10.1093/noajnl/vdaf114 (PMC12365898; doi:10.1093/noajnl/vdaf114)
Supplement: vdaf114_suppl_Supplementary_Tables_2-4 [file vdaf114_suppl_supplementary_tables_2-4.docx]

| **Prognostic factor** | **Characteristics** | **n** | **Mean OS (in weeks)** | **p** | **Cohen's d** | **Correlation** |
| --- | --- | --- | --- | --- | --- | --- |
| **Age** | Age < 70 years | 174 | 91.5 | < .001 | .6174 | -.314 < .001 |
|  | Age ≥ 70 years | 84 | 50.2 |  |  |  |
| **Sex** | Male | 148 | 76.3 | .624 | .0633 |  |
|  | Female | 110 | 80.4 |  |  |  |
| **Extent of resection** | Biopsy | 36 | 44.0 | < .001 | .5445 |  |
|  | Subtotal resection | 150 | 77.1 |  |  |  |
|  | Gross total resection | 64 | 100.4 |  |  |  |
| **KPS score**  **after surgery** | < 80 | 43 | 51.7 | .006 | .4188 | .283 < .001 |
|  | ≥ 80 | 134 | 81.2 |  |  |  |
| **MGMT** | Unmethylated | 118 | 69.0 | < .001 | .5529 | -.022 .858 |
|  | Methylated | 80 | 105.9 |  |  |  |
| **IDH** | Wildtype | 150 | 76.4 | .301 | .1679 |  |
|  | Mutant | 7 | 101.8 |  |  |  |

**Suppl. table 1:** Influence of prognostic parameters on overall survival (univariate analysis, ANOVA)

| **Prognostic factor** | **Characteristics** | **n** | **Mean PFS (in weeks)** | **p** | **Cohen's d** | **Correlation** |
| --- | --- | --- | --- | --- | --- | --- |
| **Age** | Age < 70 years | 144 | 47.2 | .361 | .1418 | -.065 .382 |
|  | Age ≥ 70 years | 37 | 40.0 |  |  |  |
| **Sex** | Male | 106 | 42.8 | .276 | .1679 |  |
|  | Female | 75 | 49.8 |  |  |  |
| **Extent of resection** | Biopsy | 20 | 20.1 | .006 | .4917 |  |
|  | Subtotal resection | 105 | 44.6 |  |  |  |
|  | Gross total resection | 52 | 55.3 |  |  |  |
| **KPS score**  **after surgery** | < 80 | 23 | 41.1 | .593 | .0895 | .092 .302 |
|  | ≥ 80 | 104 | 46.7 |  |  |  |
| **MGMT** | Unmethylated | 87 | 37.3 | .001 | .5360 | -.071 .604 |
|  | Methylated | 65 | 54.2 |  |  |  |
| **IDH** | Wildtype | 113 | 48.1 | .626 | .0895 |  |
|  | Mutant | 6 | 58.0 |  |  |  |

**Suppl. table 2:** Influence of prognostic parameters on progression-free survival (univariate analysis, ANOVA)

| **Prognostic factor** | **F** | **p** | **Cohen's d** |
| --- | --- | --- | --- |
| **Age** | 6.099 | .015 | .3920 |
| **Extent of resection** | 8.358 | .000 | .6480 |
| **MGMT** | 6.680 | .011 | .4082 |
| **Infiltration pattern** | .927 | .398 | .2109 |
| **Age * Extent of resection** | .772 | .464 | .201 |
| **Age * MGMT** | .017 | .896 | .0208 |
| **Age * Infiltration pattern** | .108 | .898 | .0633 |
| **Extent of resection * MGMT** | .840 | .434 | .201 |
| **Extent of resection * Infiltration pattern** | .322 | .863 | .1796 |
| **MGMT * Infiltration pattern** | .328 | .721 | .1267 |
| **Age * Extent of resection * MGMT** | 1.140 | .322 | .2383 |
| **Age * Extent of resection * Infiltration pattern** | .671 | .613 | .263 |
| **Age * MGMT * Infiltration pattern** | .110 | .896 | .0633 |
| **Extent of resection * MGMT * Infiltration pattern** | .487 | .745 | .2204 |
| **Age * Extent of resection * MGMT * Infiltration pattern** | .358 | .550 | .0895 |

**Suppl. table 3:** Relation of prognostic parameters with infiltration patterns (multivariate analysis, ANOVA)

| **Prognostic factor** | **F** | **p** | **Cohen's d** |
| --- | --- | --- | --- |
| **Age** | 13.527 | < .001 | .536 |
| **Extent of resection** | 18.754 | < .001 | .6328 |
| **MGMT** | 20.556 | < .001 | .663 |
| **Infiltration pattern** | 1.932 | 0.148 | .2857 |

**Suppl. table 4:** Relation of infiltration pattern to age, extent of resection, and MGMT promoter methylation status as covariates (multivariate analysis, ANOVA)
