# Supplementary material for: MRI-defined patterns of infiltration and outcome in patients with glioblastoma
Source: Neurooncol Adv. 2025 Jul 11;7(1):vdaf114. doi: 10.1093/noajnl/vdaf114 (PMC12365898; doi:10.1093/noajnl/vdaf114)
Supplement: vdaf114_suppl_Supplementary_Tables_1_Figures_2 [file vdaf114_suppl_supplementary_tables_1_figures_2.docx]

**Suppl. table 1:** Influence of prognostic parameters on overall survival (univariate analysis, ANOVA)

**Supplementary figure 1:** Kaplan Meyer analysis (overall survival) for prognostic factors. 1A, age, 1B, sex, 1C, extent of resection, 1D, Karnofsky Performance Score before resection at diagnosis, 1E, Karnofsky Performance Score after resection at diagnosis, 1F, MGMT promoter methylation, 1G, IDH mutational status and 1H, temporal muscle thickness.
